# National Cross-Sectional Data on Ideal Cardiovascular Health Among Adults in Mongolia in 2019

**DOI:** 10.5334/gh.1122

**Published:** 2022-05-30

**Authors:** Supa Pengpid, Karl Peltzer

**Affiliations:** 1 Department of Health Education and Behavioral Sciences, Faculty of Public Health, Mahidol University, Bangkok, TH; 2 Department of Research Administration and Development, University of Limpopo, Polokwane, ZA; 3 Department of Psychology, University of the Free State, Bloemfontein, South Africa; 4 Department of Psychology, College of Medical and Health Sciences, Asia University, Taichung, TW

**Keywords:** cardiovascular health, adults, Mongolia

## Abstract

**Background:**

The study aimed to estimate the prevalence, distribution, and correlates of ideal cardiovascular health (CVH) among individuals (20–69 years) in Mongolia.

**Method:**

Cross-sectional data were analyzed from 4,516 individuals (20–69 years, mean age = 42.3 years) who participated in the Mongolia STEPS survey in 2019, who had complete measurement of CVH metrics and no history of cardiovascular disease. Ideal CVH measures included non-smoking, healthy diet, physical activity, fasting blood glucose <100 mg/dL, body mass index (<23 kg/m^2^), total cholesterol <200 mg/dL, and blood pressure <120/<80 mmHg).

**Results:**

The prevalence of five to seven ideal CVH metrics was 27.7% in 2019. Most Mongolians had ideal total cholesterol (77.4%), ideal smoking (67.8%), ideal fasting glucose (73.8%) and ideal physical activity (66.0%), while a lower prevalence was found for ideal blood pressure (42.4%), ideal body mass index (31.4%), and ideal healthy diet (1.8%). The prevalence of poor smoking, poor fruit/vegetable intake, poor fasting glucose, poor blood pressure, and poor total cholesterol was higher in men than in women, while poor physical activity was higher in women than in men, and poor BMI did not differ by sex. In the adjusted logistic regression analysis, older age, male sex, and belonging to the Khalkh ethnic group were negatively associated, and a higher number of adult household members was positively associated with meeting the ideal 5–7 CVH metrics.

**Conclusions:**

The proportion of meeting 5–7 ideal CVH metrics was moderate among adults in Mongolia. Primary and secondary prevention programmes should be strengthened to improve CVH in Mongolia, considering identified associated factors.

## Background

Globally, an ‘estimated 17.9 million people died from cardiovascular diseases (CVDs) in 2016, representing 31% of all global deaths,’ of which more than three-quarters occurred in low- and middle-income countries [1]. CVDs contribute to 40% of mortality in 2016 in Mongolia, a lower-middle income country in East Asia [2]. In the previous recent STEPS survey in Mongolia in 2013, the prevalence of self-reported ischaemic heart disease and/or stroke was 12.4%, and the prevalence of CVD risk factors included 27.1% current tobacco use, 23.5% heavy episodic drinking, 96.4% inadequate fruit and vegetable intake (<5 servings/day), 22.3% low physical activity, 34.8% overweight (25–29.9 kg/m^2^), 19.7% obesity (≥30 kg/m^2^), 27.5% hypertension and 7.6% diabetes [3]. These CVD risk factors often cluster together, increasing the risk of developing CVDs, and should be prioritized in the prevention of CVDs [4,5].

In an effort to prevent the development of CVDs, the American Heart Association (AHA) conceptualized ‘ideal cardiovascular health (CVH),’ including ‘seven ideal health behaviours and factors, including smoking, body mass index, nutritional intake, physical activity, blood pressure, blood glucose level, and total cholesterol level’ [6,7]. Using these seven metrics, the CVH status of the population can be defined as ideal (5–7 ideal metrics), intermediate (3–4 ideal metrics) or poor (0–2 ideal metrics) [8]. Having a higher number of ideal CVH metrics has been shown to be ‘protective against all-cause and CVD-related mortality risk, incident cardiovascular events, lower prevalence and incidence of non-CVD outcomes such as cancer, depression, and cognitive impairment’ [9]. Based on our review, we could not find any national data on ideal CVH in Mongolia.

Globally, mainly in high-income countries, 19.6% of participants had ideal (5–7 ideal metrics) CVH [8]. Lessor research has been done on CVH in East and South Asian low- and middle-income countries. Several studies in China found, e.g., in Shandong (18–69 years) 0.05% had all 7-ideal metrics [10], in rural Northwest China (20–80 years) 0.0% [11] and in rural China (≥35 years), 0.1% had all 7 ideal CVH metrics [12]. In a nationally representative sample in China (≥20 years), 33.0% had 5–7 ideal CVH [13]. In South Asia, in Nepal (15–69 years), 51.6% had 5–7 ideal CVH metrics [14], in semi-urban Western Nepal (≥25 years), 14.3% had 6 or 7 ideal metrics [5], and in urban India (20–75 years), ideal <0.1% had 7 ideal metrics and 7.1% had ≥6 ideal metrics [15]. Worldwide, smoking (69.1%), fasting blood glucose (FPG) (67.7%) and total cholesterol (TC) (51.7%) had the highest prevalence of ideal CVH status, followed by physical activity (40.6%), body mass index (BMI) (40.3%), blood pressure (BP) (34.6%), and dietary pattern (12.1%) [8]. Inconclusive results on an increase or decrease in the prevalence of 5–7 ideal CVH metrics were found over time [16,17,18,19,20].

Sociodemographic factors associated with ideal CVH may include female sex [8,14,20], younger age [8,14,15,20,21], ethnicity [20], higher education [10,20,21], and rural residence [22,23]. The study aimed to estimate the prevalence and associated factors of ideal CVH among individuals (≥20 years) in Mongolia in 2019.

## Methods

### Study design and participants

Secondary data from the STEPS cross-sectional survey in Mongolia in 2019 with complete measurements of CVH metrics and without CVD history were analyzed [24]; the overall response rate was 98.1% [25]. Following the STEPS survey procedures, ‘Socio-demographic and behavioural information was collected in Step 1. Physical measurements such as height, weight, and blood pressure were collected in Step 2. Biochemical measurements were collected to assess blood glucose and cholesterol levels in Step 3’ [24]. ‘A multi-stage stratified sampling process was carried out to randomly select participants from the target population (15–69 years). One individual within the age range of the survey’ (15–69 years) was selected per household [25]. The inclusion criteria for the current analysis were participants with ‘no missing data on smoking status, BMI, PA, diet, total TC, FBG, and BP measurements,’ the absence of CVD and aged 20–69 years. Ethics approval was provided by the ‘Ministry of Health Medical Ethical Committee,’ Mongolia, and ‘written informed consent was obtained from all participants’ [25].

Data collection followed the ‘WHO three STEPS methodology (all assessed during one survey): Step 1 included administration of a structured questionnaire (sociodemographics, medical history, medication use, and health risk behaviour). Smoking status was sourced from three questions: 1) ‘Do you currently smoke any tobacco products, such as cigarettes, cigars or pipes?’ (Yes/No), 2) ‘In the past, did you ever smoke any tobacco products?’ and 3) ‘How old were you when you stopped smoking?’ (Age in years). Fruit and vegetable intake: Using showcards, the participants were asked about how many days they eat fruits/vegetables in a typical week and the number of servings of fruit/vegetable they eat on one of those days. Self-reported physical activity was assessed with the Global Physical Activity Questionnaire (GPAQ) and categorized by the median metabolic equivalent (METs) of performed activities as low (total physical activity METs minutes per week is < 600), moderate (3 or more days of vigorous-intensity activity of at least 20 minutes per day OR; 5 or more days of moderate-intensity activity or walking of at least 30 minutes per day OR; 5 or more days of any combination of walking, moderate or vigorous intensity activities achieving a minimum of at least 600 MET-minutes per week) and high (vigorous-intensity activity on at least 3 days achieving a minimum of at least 1500 METs minutes per week OR; 7 or more days of any combination of walking, moderate or vigorous intensity activities achieving a minimum of at least 3000 MET-minutes per week.) [25].

Step 2 consisted of blood pressure and anthropometric measurements, and Step 3 included biochemical tests (blood glucose and blood lipids) [24]. Anthropometric measurements were taken using the ‘Somatometre-Stanley 04-116 device and electronic scales GIMA’ [25]. Of the three blood pressure measurements using ‘OMRON Model M5 automatic blood pressure monitor’ [25]; the last two readings were averaged [24]. ‘Blood glucose, total cholesterol and triglycerides were measured in peripheral (capillary) blood at the data collection site using dry chemical methods, biochemical analysis and automated analyzer.’ Serum samples were taken to analyze LDL and HDL cholesterol [25]. Levels of sodium and creatinine in spot urine samples were used to estimate population 24 h salt/sodium intake, using the INTERSALT equation [25].

## Measures

Poor, intermediate and ideal CVH levels for smoking, BMI, PA, diet, TC, BP, and FBG were determined, based on modified AHA definitions; exact AHA classifications are given in brackets [6,7].

### Cardiovascular health behaviour

*Smoking status*: ≥20 years, poor: current smoker (in the past 12 months), intermediate: former smoker ≤12 months, and ideal: never or quit >12 months.

*Body Mass Index* (BMI) (kg/m^2^): ≥20 years, BMI is defined ‘poor if ≥25 kg/m^2^, intermediate as 23.0–24.9 kg/m^2^, and ideal BMI is <23.0 kg/m^2^’ [26] [‘BMI is defined poor if ≥30 kg/m^2^, intermediate as 25.0–29.9 kg/m^2^, and ideal BMI is <25 kg/m^2^’].

*Healthy diet*: adults ≥20 years, poor: <4.5 FV servings/day and ≥1500 milligrams/day sodium, intermediate: ≥4.5 FV servings/day or <1500 milligrams/day sodium, ideal: ≥4.5 FV servings/day and <1500 milligrams/day sodium [17,27]; [‘poor: 0–1 components, intermediate: 2–3, and ideal: 4–5 components (1: ≥4.5 cups/day fruits and vegetables, 2: ≥3.5 ounce servings/week of fish, 3: <1500 milligrams/day sodium, 4: <450 calories/week sweets/sugar, and 5: ≥3 1-ounce servings/day whole grains)’].

*Physical activity* (PA): adults ≥20 years, Poor = <600 MET mins/week, intermediate = 600–<1500 MET mins/week, and ideal ≥1500 MET mins/week, based on the Global Physical Activity questionnaire [17,28]. [**‘**Poor = None, Intermediate = 1–149 min/wk moderate intensity or 1–74 min/wk vigorous intensity or 1–149 min/wk moderate+vigorous, ideal = ≥150 min/wk moderate intensity or ≥75 min/wk vigorous intensity or ≥150 min/wk moderate+vigorous.’]

### Cardiovascular health factors

*Poor total cholesterol* (TC): ≥20 years, ‘poor is TC ≥6.3 mmol/L (≥240 mg/dl), intermediate is TC 5.2-6.2 mmol/L (200–239 mg/dL) or treated to TC <5.2 mmol/L (<200 mg/dL) and ideal TC is <200 mg/dL and without any cholesterol-lowering medication.’

*Fasting blood glucose (FBG)*: adults ≥20 years, poor FBG is defined as ‘glucose≥ 7.0 mmol/L (≥126 mg/dL), intermediate is glucose 5.6-6.9 mmol/L (100–125 mg/dL) or treated to <100 mg/dL, and ideal is <5.6 mmol/L <100 mg/dL and without any glucose-lowering medication.’

*Blood pressure* (BP): adults ≥20 years, poor is defined as ‘BP ≥140/≥90 mmHg, intermediate is systolic BP 120–139 mmHg or diastolic BP 80–89 mmHg or treated to BP <120/<80 mmHg, and ideal BP is defined as BP <120/<80 mmHg and without any antihypertensive medication.’

The seven CVH metrics were coded as 1 = ideal and 0 = not ideal, summed, and classified into 0–2, 3–4, and 5–7 ideal CVH metrics.

Sociodemographic covariates included age (years), sex (male, female), education in years, number of adult household members, employment and residence status and ethnic group [24].

History of CVDs included self-reported ‘Have you ever had a heart attack or chest pain from heart disease (angina) or a stroke (cerebrovascular accident or incident)? (Yes, No)’ [24].

## Data analysis

All statistical analyses were conducted with ‘STATA software version 14.0 (Stata Corporation, College Station, TX, USA).’ ‘Analysis weights were calculated by taking the inverse of the probability of selection of each participant. These weights were adjusted for differences in the age-sex composition of the sample population as compared to the target population’ [25]. Descriptive statistics are used to describe CVH metrics (ideal, intermediate, and poor). Chi-square tests were used to test for differences in proportion. Logistic regressions were used to assess the associations between sociodemographic factors and meeting 5–7 CVH metrics, overall and stratified by sex. Covariates in the multivariable logistic regression models were age group, sex, educational level, number of adult household members, work and residence status and ethnicity. To account for the multi-stage sample design, Taylor linearization methods were utilized. P-values <0.05 were considered significant, and missing values were discarded.

## Results

### Sample characteristics

The sample with complete CVH metrics measurement and without CVD history included 4,516 persons (20–69 years), with a mean age of 42.3 years (SD = 12.8 years) in 2019. Further sociodemographic characteristics of the sample by sex are described in Table 1 (see Table 1).

**Table 1:** Sample characteristics of participants aged 20 years and older, Mongolia, 2019.


	ALL	MALE	FEMALE

	N (%)	N (%)	N (%)

ALL	4516^a^	2306^b^	2480^c^

Age (years)			

20–34	1462 (32.4)	692 (34.0)	770 (31.0)

35–49	1654 (36.6)	758 (37.2)	896 (36.1)

50–69	1400 (31.0)	586 (28.8)	814 (32.8)

Education (in years)			

0–9	1305 (28.9)	708 (34.8)	597 (24.1)

10–11	1136 (25.2)	525 (25.8)	611 (24.6)

≥12	2075 (45.9)	803 (39.4)	1272 (51.3)

Household adult members			

0–2	1860 (56.1)	883 (43.8)	977 (39.6)

3 or more	2623 (43.9)	1135 (56.2)	1488 (60.4)

Employed			

No	1526 (33.8)	558 (27.4)	968 (39.1)

Yes	2984 (66.2)	1475 (72.6)	1509 (60.9)

Ethnic group			

Other	655 (14.6)	335 (16.5)	320 (13.0)

Khalkh	3844 (85.4)	1693 (83.5)	2151 (87.0)

Residence			

Rural	1644 (36.4)	821 (40.3)	823 (33.2)

Urban	2872 (63.6)	1215 (59.7)	1657 (66.8)


^a^ 879 (16.3%) were excluded due to history of CVD; ^b^ 347 (14.6%) and ^c^ 532 (17.7%) were excluded due to history of CVD.

### Distribution of cardiovascular health metrics

Most Mongolians had ideal total cholesterol (77.4%), ideal smoking (67.8%), ideal fasting glucose (73.8%) and ideal physical activity (66.0%), while a lower prevalence was found for ideal blood pressure (42.4%), ideal body mass index (31.4%) and ideal fruit/vegetable intake (1.8%). The prevalence of poor smoking, poor fruit/vegetable intake, poor fasting glucose, poor blood pressure, and poor total cholesterol was higher in men than in women, while poor physical activity was higher in women than in men, and poor BMI did not differ by sex.

The prevalence of five to seven ideal CVH metrics 27.7%, 36.4% among women and 19.3% among men (see Table 2).

**Table 2:** Cardiovascular health (CVH) metrics distribution in percent, overall and by sex.


CARDOIOVASCULAR HEALTH METRICS	ALL	MALE	FEMALE	*P-VALUE*

Smoking				<0.001

Poor	26.3	47.0	5.1	

Intermediate	5.9	9.5	2.2	

Ideal	67.8	43.5	92.7	

Body mass index				0.483

Poor	53.8	52.5	55.0	

Intermediate	14.8	15.7	13.9	

Ideal	31.4	31.7	31.1	

Healthy diet score				<0.001

Poor	70.7	74.3	67.0	

Intermediate	27.5	24.5	30.5	

Ideal	1.8	1.2	2.5	

Physical activity				<0.001

Poor	23.4	21.3	25.7	

Intermediate	10.6	7.5	13.7	

Ideal	66.0	71.2	60.6	

Total cholesterol				<0.001

Poor	4.7	5.7	3.8	

Intermediate	17.9	19.8	15.9	

Ideal	77.4	74.5	80.3	

Blood pressure				<0.001

Poor	21.9	24.4	19.4	

Intermediate	35.7	42.5	28.7	

Ideal	42.4	33.1	51.8	

Fasting glucose				<0.001

Poor	8.5	10.0	7.0	

Intermediate	17.7	19.2	16.1	

Ideal	73.8	70.8	76.9	

No of ideal CVH metrics				<0.001

0	0.9	1.4	0.3	

1	5.2	8.3	2.1	

2	15.5	20.5	10.4	

3	26.0	28.0	23.9	

4	24.6	22.4	26.9	

5	20.2	15.3	25.2	

6	7.4	4.0	10.9	

7	0.2	0.0	0.3	

≥5 ideal CVH metrics	27.7	19.3	36.4	<0.001

	**M (SD)**	**M (SD)**	**M (SD)**	

0–7 ideal CVH metrics	3.6 (1.4)	3.2 (1.3)	4.0 (1.3)	<0.001

0–14 CVH metrics	8.5 (2.3)	7.9 (2.3)	9.1 (2.2)	<0.001


Analysing CVH metrics by age group found that BMI, physical activity, total cholesterol, blood pressure and fasting glucose CVH metrics significantly declined with older age, while smoking and healthy diet did not change with age. The proportion five to seven ideal CVH metrics significantly declined from 42.3% at age 29–34 years to 10.2% at 50–69 years (see Table 3).

**Table 3:** Cardiovascular health (CVH) metrics distribution in percent, by age group.


CARDIOVASCULAR HEALTH METRICS	AGE GROUP IN YEARS			*P-VALUE*

	20–34	35–49	50–69	

Smoking				0.151

Poor	24.5	27.1	29.3	

Intermediate	6.2	5.9	5.4	

Ideal	69.3	67.0	65.2	

Body mass index				<0.001

Poor	39.3	64.4	68.7	

Intermediate	14.9	16.0	12.9	

Ideal	45.8	19.6	18.4	

Healthy diet score				0.168

Poor	69.5	72.7	70.4	

Intermediate	29.1	25.5	27.0	

Ideal	1.4	1.8	2.6	

Physical activity				<0.001

Poor	19.4	24.9	29.9	

Intermediate	9.7	11.3	11.5	

Ideal	70.8	63.8	58.6	

Total cholesterol				<0.001

Poor	3.2	5.6	6.8	

Intermediate	12.0	20.2	26.9	

Ideal	84.8	74.1	66.2	

Blood pressure				<0.001

Poor	8.7	25.1	46.0	

Intermediate	35.0	38.2	33.3	

Ideal	56.3	36.6	20.7	

Fasting glucose				<0.001

Poor	5.6	9.6	13.2	

Intermediate	13.8	20.1	22.4	

Ideal	80.6	70.4	64.4	

No of ideal CVH metrics				<0.001

0	0.6	0.6	1.9	

1	2.3	6.3	10.1	

2	10.1	17.9	23.5	

3	18.9	31.8	32.1	

4	25.9	24.4	22.3	

5	28.6	15.7	8.8	

6	13.3	3.2	1.3	

7	0.3	0.1	0.0	

≥5 ideal CVH metrics	42.3	19.0	10.2	<0.001

	**M (SD)**	**M (SD)**	**M (SD)**	

0–7 ideal CVH metrics	4.1 (1.3)	3.3 (1.2)	2.9 (1.2)	<0.001

0–14 CVH metrics	9.4 (2.2)	8.0 (2.1)	7.3 (2.2)	<0.001


### Associations with meeting 5–7 ideal CVH metrics

In the adjusted logistic regression analysis, older age, male sex, and belonging to the Khalkh ethnic group were negatively associated, and a higher number of adult household members was positively associated with meeting the ideal 5–7 CVH metrics. In addition, in the gender stratified analysis, among men, employment was negatively associated with meeting 5–7 ideal CVH metrics (see Tables 4 and 5).

**Table 4:** Associations between sociodemographic factors and 5–7 ideal CVH metrics.


VARIABLE	UNADJUSTED OR (95% CI)	ADJUSTED OR (95% CI)

Age (years)		

20–34	1 (Reference)	1 (Reference)

35–49	0.32 (0.26, 0.39)***	0.28 (0.23, 0.35)***

50–69	0.15 (0.12, 0.20)***	0.13 (0.10, 0.17)***

Gender		

Female	1 (Reference)	1 (Reference)

Male	0.42 (0.34, 0.50)***	0.36 (0.29, 0.43)***

Education (in years)		

0–9	1 (Reference)	1 (Reference)

10–11	1.01 (0.77, 1.32)	0.81 (0.61, 1.08)

≥12	1.61 (1.28, 2.03)***	0.86 (0.66, 1.11)

Household adult members		

0–2	1 (Reference)	1 (Reference)

3 or more	1.18 (0.99, 141)	1.24 (1.03, 1.50)*

Employed		

No	1 (Reference)	1 (Reference)

Yes	0.78 (0.65, 0.94)**	0.85 (0.68, 1.07)

Ethnic group		

Other	1 (Reference)	1 (Reference)

Khalkh	0.72 (0.54, 0.94)*	0.72 (0.53, 0.96)*

Residence		

Rural	1 (Reference)	1 (Reference)

Urban	1.06 (0.86, 1.31)	1.09 (0.85, 1.38)


OR = Odds Ratio; CI = Confidence Intervals; * *p* < 0.05; ** *p* < 0.01; *** *p* < 0.001.

**Table 5:** Adjusted associations between sociodemographic factors and 5–7 ideal CVH metrics by sex.


VARIABLE	UNADJUSTED OR (95% CI)	ADJUSTED OR (95% CI)

**Male**		

Age (years)		

20–34	1 (Reference)	1 (Reference)

35–49	0.25 (0.18, 0.38)***	0.25 (0.18, 0.36)***

50–69	0.17 (0.11, 0.26)***	0.14 (0.09, 0.22)***

Education (in years)		

0–9	1 (Reference)	1 (Reference)

10–11	1.01 (0.66, 1.55)	0.74 (0.47, 1.16)

≥12	1.45 (0.99, 2.12)	0.83 (0.54, 1.27)

Household adult members		

0–2	1 (Reference)	1 (Reference)

3 or more	1.55 (1.16, 2.05)**	1.54 (1.15, 2.08)**

Employed		

No	1 (Reference)	1 (Reference)

Yes	0.56 (0.40, 0.78)***	0.55 (0.38, 0.80)**

Ethnic group		

Other	1 (Reference)	1 (Reference)

Khalkh	0.59 (0.38, 0.91)*	0.74 (0.48, 1.12)

Residence		

Rural	1 (Reference)	1 (Reference)

Urban	0.93 (0.66, 1.32)	1.11 (0.76, 1.61)

**Female**		

Age (years)		

20–34	1 (Reference)	1 (Reference)

35–49	0.31 (0.24, 0.39)***	0.29 (0.22, 0.38)***

50–69	0.12 (0.09, 0.17)***	0.12 (0.09, 0.17)***

Education (in years)		

0–9	1 (Reference)	1 (Reference)

10–11	0.90 (0.64, 1.28)	0.85 (0.60, 1.20)

≥12	1.43 (1.07, 1.91)*	0.82 (0.60, 1.14)

Household adult members		

0–2	1 (Reference)	1 (Reference)

3 or more	1.04 (0.83, 1.31)	1.04 (0.82, 1.32)

Employed		

No	1 (Reference)	1 (Reference)

Yes	1.14 (0.91, 1.42)	1.17 (0.90, 1.51)

Ethnic group		

Other	1 (Reference)	1 (Reference)

Khalkh	0.74 (0.55, 0.99)*	0.72 (0.52, 1.01)

Residence		

Rural	1 (Reference)	1 (Reference)

Urban	1.06 (0.83, 1.35)	1.10 (0.83, 1.46)


OR = Odds Ratio; CI = Confidence Intervals; * *p* < 0.05; ** *p* < 0.01; *** *p* < 0.001.

## Discussion

The study presents for the first-time national data on the prevalence and distribution of CVH metrics in nationally representative samples of adults in Mongolia in 2019. The found prevalence of ideal CVH (5–7 ideal metrics) (27.7%), was higher than global estimates, mainly in high-income countries, ideal CVH (having 5–7 ideal metrics) (19.6%) CVH [8], but lower than in China (33.0%, 5–7 ideal metrics) [13], and in Nepal (51.6%, 5–7 ideal metrics) [14]. The proportion of ideal CVH metrics (all 7 metrics) (0.2%), was similar to in urban India (<0.1% had 7 ideal metrics) [15], Shandon in China (0.05% all 7-ideal metrics) [10], and in rural China (0.0% all 7-ideal metrics) [11].

Like the three best and lowest global estimates [8], this investigation found the highest ideal CVH status for TC (77.4%), smoking (67.8%), and FGP (73.8%), and the lowest for healthy diet (1.8%). The proportion of ideal fruit and vegetable consumption (21.8%) compares with global rates in low- and middle-income countries (18.0%) [29]. The prevalence of ideal PA (66.0%) was higher than globally (40.6%), and the proportion of ideal BMI (31.4%) was lower than global figures (40.3%) [8]. The proportion of poor blood pressure or hypertension (21.9%) was still slightly higher compared to global estimates in lower-resource countries (17.5%) [30]. Previous research in Mongolia, also found a high prevalence of poor diet and overweight among adults calling for expanded supplementation and food fortification to address micronutrient inadequacies [31].

Consistent with previous research [8,14,15,20,21], the ideal CVH was higher among younger age groups and among women. The overall better performance of women than men on ideal CVH may be largely explained by their high proportion of ideal smoking, ideal healthy diet, ideal blood pressure, ideal total cholesterol and ideal FBG compared to men. Therefore, men should be particularly targeted, regarding tobacco, blood pressure, cholesterol and glucose control as well as healthy diet, and women regarding physical activity. Some previous research [10,20,21,22,23] showed an association between higher education, rural residence and ideal CVH metrics, while we did not find significant differences in this regard.

Higher number of adult household members (as a proxy for lower economic status) was associated with higher ideal CVH metrics in this study, which is consistent with a study in Uganda that found an association between lower income and higher ideal CVH metrics [21], while in China higher income was associated with better ideal CVH metrics [10]. In line with some previous study that found ethnic differences in the prevalence of ideal CVH metrics [20], we found that belonging to the main ethnic group (Khalkh) decreased the odds of ideal CVH metrics.

Study results may inform the national non-communicable diseases (NCD) policy in Mongolia. CVH interventions should include multiple strategies including changes in health care policy aiming at promoting health behaviour, the reinforcement of individual health behaviour, and safe healthcare management [32]. For example, since 2015 the Mongolian national salt reduction strategy has been implemented by ‘creating a social, economic and legal environment that supports salt reduction, including by influencing food supply, increasing partnerships between government and relevant stakeholders, and creating an enabling environment to support improved consumer choices’ [33]. Part of this initiative included training of chefs, students and employees to implement salt reduction in schools, hospitals, workplaces and kindergartens, and voluntary reductions in salt in bread and bakery products agree on with major companies in 2011 [34]. However, the average daily salt intake in the 15–69 years-old population was 10.5 gr (more than double the WHO recommendation of <5gr) in 2019, which did not much improve compared to the 2013 survey, 11.1 gr in Mongolia [33]. Tobacco use may have reduced in Mongolia due to tobacco demand-reduction measures, and an increase of excise taxes on tobacco, but more needs to be done to initiate and control tobacco use [35,36]. Body weight and physical inactivity have increased in Mongolia, which may in part be attributed to no change on public awareness on diet and/or physical activity [35]. To improve CVH in Mongolia, more needs to be done including ‘promotion of body weight control, smoking cessation, healthy diets, and screening and control of high levels of blood sugar and blood pressure’ [35,36]. The Mongolian government in recognising the critical importance of improving CVH, primary care was seen as an important entry point to reach this goal and emphasize that a stronger health system with better resources is needed. Using the ‘MongPEN (WHO’s Package of Essential NCD interventions, commonly known as “PEN”) approach,’ the health system should be strengthened to prevent and manage NCDs, including ‘improving heart health (from WHO’s “HEARTS” technical package), such as guidance on how to identify patients at high risk of CVD, and making use of an ePrescription service to gather data’ [37].

The study strengths include the use of nationally representative sample and standardized STEPS methodology and measures. Some variables were evaluated by self-report, which may have biased responses, and the cross-sectional design precludes causative conclusions between the evaluated variables. We did not use the original five components but only two components (fruit and vegetable and sodium intake) AHA healthy diet, which should be included in further research [8,9].

## Conclusion

The proportion of meeting 5–7 ideal CVH metrics was moderate among adults in Mongolia. Primary and secondary prevention programmes should be strengthened to improve CVH in Mongolia, considering identified associated factors. Future research may want to include more comprehensive measures on a healthy diet.

## Data Accessibility Statement

The data source is publicly available at the World Health Organization NCD Microdata Repository (URL: https://extranet.who.int/ncdsmicrodata/index.php/catalog).
